# Identification of Glycolysis-Related lncRNAs and the Novel lncRNA WAC-AS1 Promotes Glycolysis and Tumor Progression in Hepatocellular Carcinoma

**DOI:** 10.3389/fonc.2021.733595

**Published:** 2021-08-30

**Authors:** Xigang Xia, Hao Zhang, Peng Xia, Yimin Zhu, Jie Liu, Kequan Xu, Yufeng Yuan

**Affiliations:** ^1^Department of Hepatobiliary and Pancreatic Surgery, Zhongnan Hospital of Wuhan University, Wuhan, China; ^2^Clinical Medicine Research Center for Minimally Invasive Procedure of Hepatobiliary & Pancreatic Diseases of Hubei Province, Wuhan, China

**Keywords:** hepatocellular carcinoma, glycolysis, glycolysis-related lncRNAs, prognosis, WAC-AS1, ARPP19, miR-320d

## Abstract

**Background:**

High glycolysis efficiency in tumor cells can promote tumor growth. lncRNAs play an important role in the proliferation, metabolism and migration of cancer cells, but their regulation of tumor glycolysis is currently not well researched.

**Methods:**

We analyzed the co-expression of glycolysis-related genes and lncRNAs in The Cancer Genome Atlas (TCGA) database to screen glycolysis-related lncRNAs. Further prognostic analysis and differential expression analysis were performed. We further analyzed the relationship between lncRNAs and tumor immune infiltration. Since WAC antisense RNA 1 (WAC-AS1) had the greatest effect on the prognosis among all screened lncRNAs and had a larger coefficient in the prognostic model, we chose WAC-AS1 for further verification experiments and investigated the function and mechanism of action of WAC-AS1 in hepatocellular carcinoma.

**Results:**

We screened 502 lncRNAs that have co-expression relationships with glycolytic genes based on co-expression analysis. Among them, 112 lncRNAs were abnormally expressed in liver cancer, and 40 lncRNAs were related to the prognosis of patients. Eight lncRNAs (WAC-AS1, SNHG3, SNHG12, MSC-AS1, MIR210HG, PTOV1-AS1, AC145207.5 and AL031985.3) were used to established a prognostic model. Independent prognostic analysis (P<0.001), survival analysis (P<0.001), receiver operating characteristic (ROC) curve analysis (AUC=0.779) and clinical correlation analysis (P<0.001) all indicated that the prognostic model has good predictive power and that the risk score can be used as an independent prognostic factor (P<0.001). The risk score and lncRNAs in the model were found to be related to a variety of immune cell infiltration and immune functions. WAC-AS1 was found to affect glycolysis and promote tumor proliferation (P<0.01). WAC-AS1 affected the expression of several glycolysis-related genes (cAMP regulated phosphoprotein 19 (ARPP19), CHST12, MED24 and KIF2A) (P<0.01). Under hypoxic conditions, WAC-AS1 regulated ARPP19 by sponging miR-320d to promote glucose uptake and lactate production (P<0.01).

**Conclusion:**

We constructed a model based on glycolysis-related lncRNAs to evaluate the prognostic risk of patients. The risk score and lncRNAs in the model were related to immune cell infiltration. WAC-AS1 can regulate ARPP19 to promote glycolysis and proliferation by sponging miR-320d.

## Introduction

Hepatocellular carcinoma (HCC) is the sixth most common malignant tumor in the world and the fourth leading cause of tumor-related death, with approximately 841,000 new cases and 782,000 related deaths each year (1). HCC accounts for 75–80% of all liver cancer cases (2, 3). Although a large number of studies on HCC have led to continuous progress in the treatment of liver cancer, the long-term survival rate is still low among liver cancer patients (4). Due to the high heterogeneity and molecular diversities, the prognosis of patients with nonviral HCC is widely divergent. Currently, HCC prognostication mainly relies on clinicopathological staging and some biomarkers (5, 6). Therefore, it is very important to further explore the molecular mechanism of liver cancer development and develop new prognostic indicators.

The reprogramming of energy metabolism is an important feature of cancer cells that represents one of the “hallmarks of cancer” (7). Among them, the enhancement of glycolysis is an important part of tumor cell metabolic reprogramming (8). The enhancement of tumor glycolysis can promote the proliferation, invasion and metastasis and other malignant behaviors of various tumors (9, 10). In addition, studies have reported that the enhancement of glycolysis is related to immune cell infiltration in the tumor immune microenvironment and resistance to immunotherapy (11, 12).

Long noncoding RNAs (lncRNAs) are transcripts of more than 200 nucleotides that do not encode proteins. lncRNAs play an important role in the physiological activities of cells and can regulate the occurrence and progression of cancer in many ways, including by regulating their proliferation, invasion and metastasis (13). Increasing evidence shows that lncRNAs also play an important role in regulating tumor metabolism (such as glucose metabolism and fat metabolism), immune tolerance, and immune infiltration in the tumor microenvironment (14–16). Because of their important physiological functions, lncRNAs can be used as effective tumor prognostic markers, and research on their regulatory mechanism has great significance (17).

In this study, we screened lncRNAs related to glycolysis-related genes (GR genes) through co-expression analysis. Then, univariate Cox regression analysis was used to further screen out prognosis-related lncRNAs. We used least absolute shrinkage and selection operator (LASSO) regression analysis to construct a prognostic model based on 8 lncRNAs (WAC-AS1, SNHG3, SNHG12, MSC-AS1, MIR210HG, PTOV1-AS1, AC145207.5 and AL031985.3). Survival analysis and receiver operating characteristic (ROC) curve analysis were used to assess its predictive power. Immune infiltration analysis was used to study the relationship between the risk score and immune cell infiltration. For the novel lncRNA WAC-AS1, we verified the abnormally high expression of WAC-AS1 in liver cancer through experiments and found that it promotes glycolysis in liver cancer cells and regulates a variety of GR genes. Bioinformatics prediction and dual luciferase reporter assays were used to validate that WAC-AS1 can affect the expression of ARPP19 by sponging miR-320d.

In short, we screened glycolysis-related lncRNAs (GR lncRNAs) based on the expression of GR genes and lncRNAs and constructed a prognostic model based on 8 lncRNAs that can be used to predict the long-term survival rate of patients with HCC. The prognostic model has good predictive power. WAC-AS1 regulates a variety of GR genes, which can promote the glycolysis efficiency of liver cancer cells and promote the proliferation of liver cancer cells. We predicted and validated that WAC-AS1 regulates ARPP19 by sponging miR-320d with *in vitro* experiments.

## Materials and Methods

### Data Collection

The clinical data and high-throughput sequencing data of 424 patients were obtained from The Cancer Genome Atlas (TCGA) database, including the expression levels of mRNA and lncRNAs. The GRgene set, which contains 200 genes (M5937), was obtained from the official GSEA website (gsea-msigdb.org/gsea). The expression levels of lncRNAs and GR genes were extracted from the TCGA data, and then a correlation test was performed to screen out lncRNAs that have a co-expression relationship with GR genes using the “limma” R package. The threshold value of the correlation coefficient was set to 0.4, and the P value was required to be less than 0.01. The datasets (GSE14520) downloaded from the GEO database (www.ncbi.nlm.nih.gov/geo/) were used for validation. The tissues of 62 patients who underwent liver tumor resection during 2013-2015 were obtained from the Biological Repositories of Hepatobiliary and Pancreatic Surgery, Zhongnan Hospital of Wuhan University. All patients in this cohort underwent curative hepatectomy. The resected samples of all patients were confirmed by pathological examination. The study was approved by the Ethics Committee of Zhongnan Hospital, and the methods were carried out in accordance with the approved guidelines. All patients signed informed consent forms agreeing that their specimens could be used for future scientific research before their samples were included in the biological repositories.

### Identified Differentially Expressed lncRNAs and Prognosis-Related lncRNAs

Based on the sequencing data of 374 tumor tissues and 50 normal liver tissues, difference analysis was performed using the “limma” R package. The abnormally expressed lncRNAs in liver cancer tissues (false discovery rate (FDR)<0.05, log2fold change (log2(FC)>1) were screened out. This approach was also used for screening differentially expressed miRNAs. According to the relationship between the expression of lncRNAs and the survival time and survival status of patients, univariate Cox regression analysis was performed to screen out prognosis-related lncRNAs (P<0.01).

### Construction of Prognostic Models

According to the previous analysis, the results of the analysis were intersected to reveal GR lncRNAs that are abnormally expressed and related to patient overall survival. The “Venn” R package and the “pheatmap” package were used to draw the Venn diagram and the heatmap, respectively. According to the expression of lncRNAs and the survival time of the patient, the “glmnet” R package was used to establish the prognosis model through LASSO regression analysis and risk score calculation for each patient. Survival analysis, ROC curve analysis and other methods were applied to test the predictive power of the model. The regulatory relationship between lncRNAs and GR genes in the model was visualized by drawing a network diagram with Cytoscape software (Version 3.7.1).

### Verification of the Prognostic Model

The model was tested with a variety of methods. According to the risk score, patients were divided into a high-risk group and a low-risk group, and then the “survminer” R package was used to draw the survival curves of the patients and calculate the survival difference. Risk curves based on the patients’ risk scores showed clear changes in survival time and survival status as the risk score increased. The “survivalROC” R package was used to draw the ROC curve and calculate the area under the curve (AUC), which reflects the predictive power of the model. The GSE14520 dataset from the GEO database was used as an independent validation cohort (n = 203).

### Independent Prognostic Analysis and Principal Component Analysis

To judge whether the risk score is an independent prognostic factor, the patient’s age, sex, disease stage, tumor grade and risk score were all included as variables for the multivariate Cox regression analysis. Hazard ratios and 95% confidence intervals were calculated for all factors. When the hazard ratio>1 and the P value<0.05, the variable was considered to be an independent prognostic factor affecting the patient. Then, clinical correlation analysis of the risk score was performed. The patients were divided into groups according to their age, sex, disease stage, and tumor grade. In different groups, the survival curves of high-risk and low-risk patients were drawn, and the survival difference was calculated. According to the results, it was judged whether the risk score was related to clinical factors such as the patient’s age and sex. Then, PCA was performed with “Rtsne” R package.

### Gene Set Enrichment Analysis

Through enrichment analysis, the biological function of the molecule can be predicted. GSEA was conducted using GSEA v4.0.2 software (https://www.gsea-msigdb.org/gsea/index.jsp). The patients were divided into two groups according to the risk score and the median expression of lncRNAs in the prediction model. During the analysis, the Kegg v7.4 symbol gene set was used. The number of permutations was set to 1,000. The enrichment maps were output, and the P values and FDR values were calculated.

### Immune Infiltration Analysis

The level of immune cell infiltration in the tumor immune microenvironment is related to the level of tumor cell gene expression, and studies have provided a quantitative method to calculate the level of immune cell infiltration (18). Therefore, we used single-sample gene set enrichment analysis (ssGSEA) to quantify the infiltration level of 22 immune cells in the tumor immune microenvironment. The infiltration level of each immune cell is shown by the enrichment score of ssGSEA. Patients were grouped according to different factors, and the difference in immune cell infiltration was calculated to study whether there was an association between grouping factors and immune cell infiltration.

### Cell Culture and Transfection

Normal LO2 hepatocytes, 293T cells and HCC cell lines (Huh7, HepG2, Hep3B, SNU387 and HCCLM3) were obtained from Shanghai Cell Bank (Shanghai, China). Hep3B cells were grown in MEM (Invitrogen, Carlsbad, CA), and other cell lines were grown in DMEM (Invitrogen) supplemented with 10% fetal bovine serum (Invitrogen). Jtsbio (Guangzhou, China) constructed and synthesized siRNAs specific for WAC-AS1 as follows:

WAC-AS1-siRNA 1-F: 5’-AGGAGAAGAAAGAGAGAAATT -3’;
WAC-AS1-siRNA 1-R: 5’-UUUCUCUCUUUCUUCUCCUTT -3’.
WAC-AS1-siRNA 3-F: 5’-GGAAAUGGGGAAAGAAUAATT-3’;
WAC-AS1-siRNA 3-R: 5’-UUAUUCUUUCCCCAUUUCCTT-3’.

A WAC-AS1 overexpressing plasmid was constructed using the pcDNA3.1 vector (Invitrogen). The empty vector was used as a control. miR-320d mimics and inhibitors were synthesized by Tsingke (Wuhan, China). Transfection of plasmid DNA, miR-320d mimics and inhibitor was conducted using Lipofectamine 3000 reagent (Invitrogen).

### RNA Extraction and RT-qPCR Analysis

Total RNA from liver tissues was extracted by TRIzol (Invitrogen) according to its protocol. Total RNA was also extracted using TRIzol reagent and converted into cDNA using the PrimeScript RT kit (TaKaRa, Dalian, China). Then, RT-qPCR was performed using SYBR Green Master Mix (Applied Biosystems, Foster City, CA, USA) with the ABI PRISM 7900 Sequence Detection System (Applied Biosystems). The PCR cycling was as follows: pre-denaturation at 95 °C for 10 min, followed by 40 cycles of denaturation at 95 °C for 10 s, annealing at 55 °C for 25 s and extension at 72 °C for 10 s. Data were analyzed using the relative quantification (2−ΔΔCT) method, and β-actin was used as a reference gene for mRNA quantification. The primer sequences were as follows (others are shown in **Table S1**):

WAC-AS1-F: 5’-CCTGCCCACCCTCTCTTTATC-3’;
WAC-AS1-R: 5’-AGTGGAGTAGACAAGGACGAC-3’;
miR-320d-F: 5′-AAAAGCTGGGTTGAGAGGA-3′;
miR-320d-R: 5′-TCCTCTCAACCCAGCTTTT-3′;
β-actin-F: 5’-CCTTCCTGGGCATGGAGTC-3’;
β-actin -R: 5’-TGATCTTCATTGTGCTGGGTG-3’.

### Detection of Cell Proliferation *In Vitro* and *In Vivo*

Cell viability was detected by using Cell Counting Kit-8 (CCK-8) following the manufacturer’s instructions. The spectrophotometric absorbance of each sample was measured at 450 nm using an Infinite M200 spectrophotometer (Tecan, Switzerland). After completing the designated intervention, HCC cells were cultured in 6-well plates at a density of 2,000 cells per well. After 14 days, the cells were fixed with paraformaldehyde and stained with crystal violet. A microscope was used to count the number of tumorspheres. Treated cells (3 × 106 cells for Huh7 transfected with pcDNA/WAC-AS1) and control cells (Huh7 with Vector) were subcutaneously injected into the right and left dorsal flank of 4-week-old male BALB/C nude mice (Shulaibao, Wuhan, China), respectively. The tumors formed were measured with a caliper and tumor volumes [long diameter × (short diameter)^2^ × 1/2] were calculated. After 2 weeks, subcutaneous tumors were stripped.

### Cell Cycle Assay

To assess the cell cycle, the cells were resuspended in PBS buffer and treated with a cell cycle staining kit (MultiSciences, Hangzhou, China) according to the manufacturer’s instructions. The cell cycle was analyzed by fluorescence-activated cell sorting (FACS) using flow cytometry (Beckman, USA).

### Measurement of Glucose Uptake, Lactate Production, and Extracellular Acidification Rate

Glucose uptake and lactate production were calculated by detecting the glucose content of the medium to evaluate the glycolysis efficiency of the cells. The glucose level was measured using a glucose colorimetric assay kit II (K686-100, BioVision, Milpitas, CA, USA). A lactic acid colorimetric assay kit (K627-100, BioVision) was used to determine the lactic acid level. All testing procedures were carried out in accordance with the manufacturer’s instructions. The Seahorse Extracellular Flux Analyzer XF96 (Seahorse Bioscience, www.seahorsebio.com) was used to monitor *in vitro* cells metabolic alternations, according to the manufacturer’s instructions. For detection of the real-time glycolytic rate, cells were incubated with unbuffered medium followed by a sequential injection of 10 mM glucose, 1 µM oligomycin (OM), and 80 mM 2-deoxyglucose (2-DG). Oligomycin and 2-deoxyglucose were purchased from Topscience (shanghai, China). Considering that the number of cells in each sample may be different, the concentrations of glucose or lactic acid and ECAR measurements were normalized to the cell number.

### Dual-Luciferase Reporter Assay

The target gene of miR-320d was predicted by the online tools TargetScan (19), starBase (20) and miRDB (21). The target miRNA of WAC-AS1 was predicted by starBase, miRDB and ENCORI. A dual luciferase reporter assay (RG027, Beyotime, Shanghai, China) was performed to identify whether the WAC-AS1 and ARPP19 3′-UTR sequences contained the miR-320d binding site. 293T cells were used for the dual-luciferase reporter assay. The protocol was performed in accordance with the manufacturers’ instructions.

### Western Blot Analysis

Anti-LDHA antibody (21799-1-AP), anti-TPI1 antibody (10713-1-AP), anti-ARPP19 antibody (11678-1-AP), anti-KIF2A antibody (13105-1-AP) and anti-beta actin antibody (66009-1-Ig) were purchased from Proteintech (Wuhan, China). Total proteins were extracted utilizing SDS (Beyotime, Shanghai, China). The detailed protocols for western blotting were based on previously described methods (22). A ChemiDoc MP Imaging System (Bio-Rad) was used to detect and normalize the protein expression levels according to the housekeeping gene β‐actin. The protein concentration was measured by the BCA protein assay (Cat. 23225, Thermo Fisher Scientific, Waltham, MA, USA).

### Statistical Analysis

SPSS 24.0 software (SPSS Inc., Chicago, USA) and GraphPad Prism6 (GraphPad Sofware, Inc., San Diego, CA, USA) were used for statistical analyses. The results are presented as the mean ± standard deviation (SD) of three independent experiments. Statistical analysis was performed using unpaired Student’s t-test to compare the continuous variables. To compare the categorical variables, χ2 test was performed to assess the pathological and clinical characteristics of the WAC-AS1 high/low groups. Survival time between groups was evaluated using Kaplan–Meier method and compared by log-rank test. P < 0.05 was considered to indicate significance. ∗P < 0.05; ∗∗P < 0.01; ***p < 0.001.

## Results

### Screening of DE lncRNAs Related to Patient Prognosis and Glycolysis

**Figure 1:**
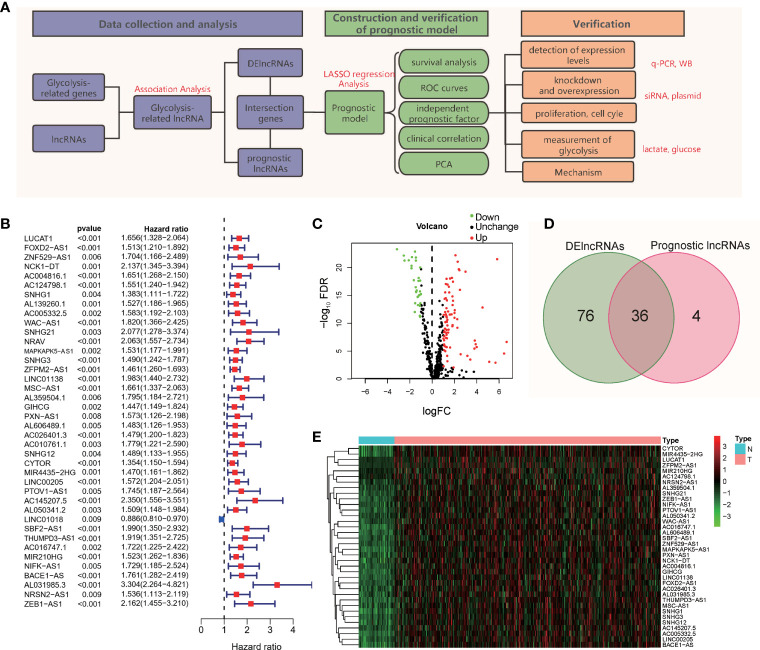
Flowchart and lncRNA screening process. **(A)** Flowchart of the study. **(B)** Forest plot of univariate Cox regression analysis; **(C)** Volcano map of differentially expressed genes; **(D)** Intersection of DE lncRNAs and prognostic lncRNAs; **(E)** Heatmap of the 36 intersecting lncRNAs.

To better show the experimental process and content of the subject, we drew a flowchart of the experiment (**Figure 1A**). As shown in the flow chart, the first step of the research is to obtain lncRNAs with potential research value. We obtained 200 gene symbols related to glycolysis on the official website of GSEA (**Table S2**). Through correlation analysis, 502 lncRNAs (**Table S3**) that have co-expression relationships with glycolytic genes were screened out, and the correlation coefficient was set to > 0.4. Based on the follow-up data and the expression levels of lncRNAs in 370 patients, excluding patients with incomplete follow-up data, 40 prognosis-related lncRNAs were screened out from the 502 lncRNAs (**Figure 1B**). According to the expression level of lncRNAs in 50 normal tissues and 374 tumor tissues, 112 abnormally expressed lncRNAs were screened out from the 502 GR lncRNAs; 81 were upregulated, and 31 were downregulated (**Figure 1C**) (log2(FC)>1, FDR< 0.01). The intersection of the results showed 36 lncRNAs (**Figure 1D**), and a heat map of these lncRNAs was drawn (**Figure 1E**). These 36 lncRNAs are abnormally expressed in tumor tissues, are related to prognosis and have a co-expression relationship with glycolytic genes.

### Construction and Verification of Prognostic Models

**Figure 2:**
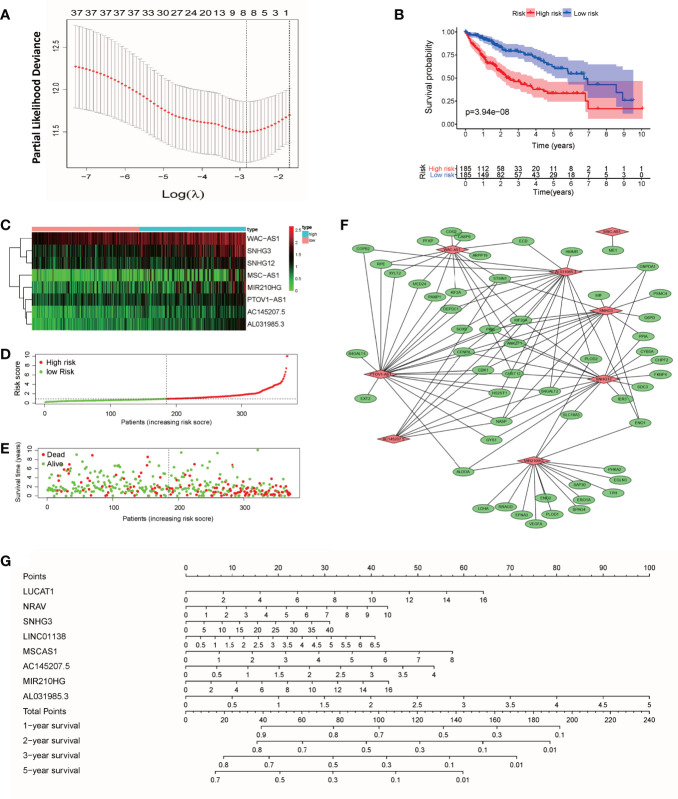
Construction of the prognostic model and nomogram. **(A)** “Leave-one-out” cross-validation for parameter selection during LASSO regression; **(B)** survival curves of patients in the high-risk and low-risk groups; **(C)** heatmap of lncRNAs in the model; **(D)** curve of the risk score; **(E)** distribution of survival status; **(F)** co-expression relationship between glycolytic genes and lncRNAs in the model; **(G)** nomogram for survival prediction.

According to the expression of lncRNAs and the prognosis and survival status of patients, LASSO regression analysis was used to establish an 8-lncRNA (WAC-AS1, SNHG3, SNHG12, MSC-AS1, MIR210HG, PTOV1-AS1, AC145207.5 and AL031985.3) prognostic model from the 36 lncRNAs (**Figure 2A** and **Figure S1**). Risk scores were calculated according to the following formula: Risk score=(Ex**p_WAC-AS1_**×0.722) + (Ex**p_SNHG3_**×0.299) + (Exp**_SNHG12_**×0.475) + (Exp**_MSC-AS1_**×0.044) + (Exp**_MIR210HG_**×0.179) + (Exp**_PTOV1-AS1_**×0.015) + (Exp**_AC145207.5_**×0.107) + (Exp**_AL031985.3_** ×0.202) (Exp, Expression). According to the median risk score, patients were divided into a high-risk group and a low-risk group. There was a significant difference in prognosis between the two groups (P<0.001) (**Figure 2B**). In the two groups of patients, the expression levels of WAC-AS1, SNHG3 and MIR210HG were significantly different, indicating that they are closely related to the risk score and are key molecules in the prognostic model (**Figure 2C**). With the increase in the risk score, the trend of survival time of patients gradually decreased, and the proportion of patients whose follow-up outcome was death gradually increased (**Figures 2D, E**), indicating that the risk score is closely related to survival time and survival status. We visualized the co-expression relationship between glycolytic genes and lncRNAs in the model (**Figure 2F**). To make the prognostic model more convenient for clinical use, we constructed a nomogram that can calculate the risk score based on the expression of lncRNAs and predict the survival rate of patients at 1, 2, 3 and 5 years (**Figure 2G**). The GSE14520 dataset from the GEO database was used as an independent validation cohort (n = 203). The ROC curve and calibration were used to assess the discriminating ability of the nomogram. ROC curve analysis (AUC=0.751) showed that the nomogram had good predictive accuracy for prognosis. Calibration plots showed excellent calibration of the nomogram (**Figure S2**).

### The Model’s Predictive Power and the Correlation Test of Clinical Factors

**Figure 3:**
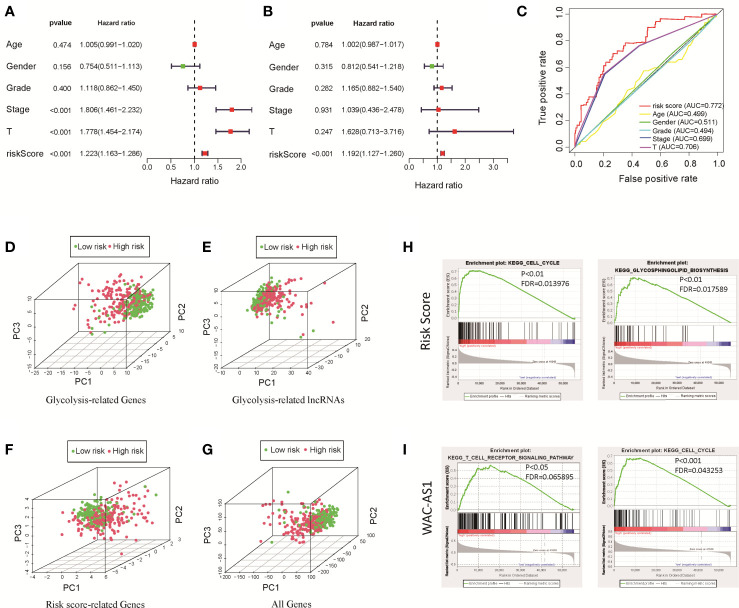
Clinical relevance analysis, PCA and GSEA analysis results for the risk score and GR lncRNAs. **(A)** Univariate Cox regression analysis of clinical factors and the risk score; **(B)** multivariate Cox regression analysis of clinical factors and the risk score; **(C)** ROC curve; **(D)** PCA results for GR genes; **(E)** PCA results for GR lncRNAs; **(F)** PCA results for risk score-related lncRNAs; **(G)** PCA results for all genes; **(H)** GSEA results for the risk score; **(I)** GSEA results for WAC-AS1.

We used univariate and multivariate Cox regression analyses to identify independent prognostic factors. Univariate Cox regression analysis revealed that risk score, disease stage and T stage were related to prognosis (**Figure 3A**). Multivariate Cox regression analysis showed that the risk score was an independent factor affecting prognosis (**Figure 3B**). We used the ROC curve to detect the predictive power of the model, and the results showed that the risk score can better reflect the prognosis of patients than disease stage and tumor grade (**Figure 3C**). The predictive accuracy of the lncRNA model was then verified in the GEO validation group (GSE14520) through survival analysis and ROC curve analysis (**Figure S3**). To clarify whether there is a correlation between the prediction model and clinical factors, we performed a set of predefined stratified analyses. In different groups, the survival curves of high-risk and low-risk patients were significantly different (**Figure S4**). In conclusion, the model predicted patient survival independently of clinical factors, including age, sex, and stage of the disease.

### Principal Component Analysis and GSEA

The results of PCA showed that between patients in the high-risk and low-risk groups, the GR genes (**Figure 3D**), GR lncRNAs (**Figure 3E**), risk score-related genes (**Figure 3F**) and all genes (**Figure 3G**) had different expression profiles. This shows that the model constructed based on 8 lncRNAs can effectively classify patients. To explore the underlying mechanism of the prognostic signature, we divided patients into two groups based on the risk score and the expression of each lncRNA in the model and performed GSEA. The results showed that in high-risk patients, genes related to glucose and lipid metabolism and the cell cycle were significantly enriched (P<0.01; **Figure 3H**). Highly expressed lncRNAs, such as SNHG12, MIR210HG and WAC-AS1, were related to the cell cycle, immune-related pathways, cancer-related pathways and carbohydrate metabolism-related pathways (**Figure 3I** and **Figure S5**). These findings indicate that there are differences in metabolism- and cell cycle-related genes between the high-risk and low-risk groups, which may partly explain the significant difference in prognosis.

### The Risk Score and Risk-Related lncRNAs Are Related to Immune Cell Infiltration

**Figure 4:**
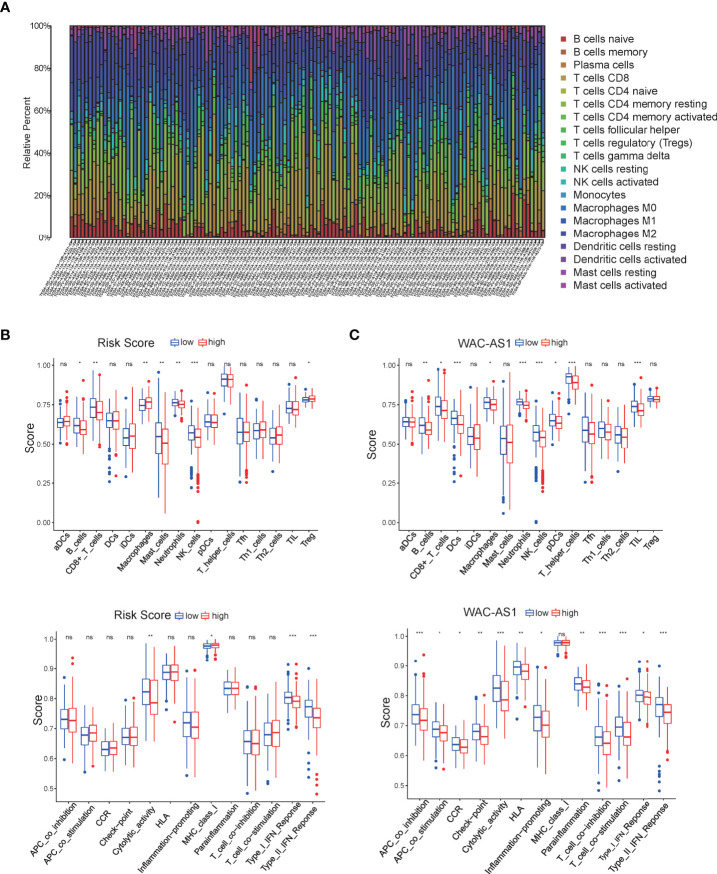
Characterization of immune cell infiltration and immune-related functions. **(A)** Bar chart of the proportions for 22 immune cell types; **(B)** relationship of the risk score with immune cell infiltration and the immune response; **(C)** relationship of WAC-AS1 with immune cell infiltration and the immune response. ∗P < 0.05, ∗∗P < 0.01, ***p < 0.001; ns, no significance.

The ssGSEA algorithm was used to evaluate the infiltration status of immune cells in the TCGA cohort. The proportions of 22 immune cells in each patient are shown in **Figure 4A**. Subsequently, the CIBERSORT algorithm was used to study immune infiltration in the liver cancer microenvironment between high-risk and low-risk patients. The results showed that macrophages were positively correlated with risk scores, and CD8 T cells, mast cells, NK cells and IFN immune responses were negatively correlated with the risk score, indicating that the poor prognosis of high-risk patients may be related to immune cell infiltration and the immune response (**Figure 4B**). We further carried out immune infiltration analysis of lncRNAs related to the risk score. The results showed that MSC-AS1, WAC-AS1 and SNHG12 were associated with CD8 T cell infiltration; AL031985.3, MSC-AS1, SNHG12 and WAC-AS1 were related to macrophage infiltration; and MSC-AS1, SNHG12 and WAC-AS1 were significantly related to immune checkpoints (**Figure 4C** and **Figure S6**).

### WAC-AS1 Is Highly Expressed in Liver Cancer and Associated With Poor Prognosis

**Figure 5:**
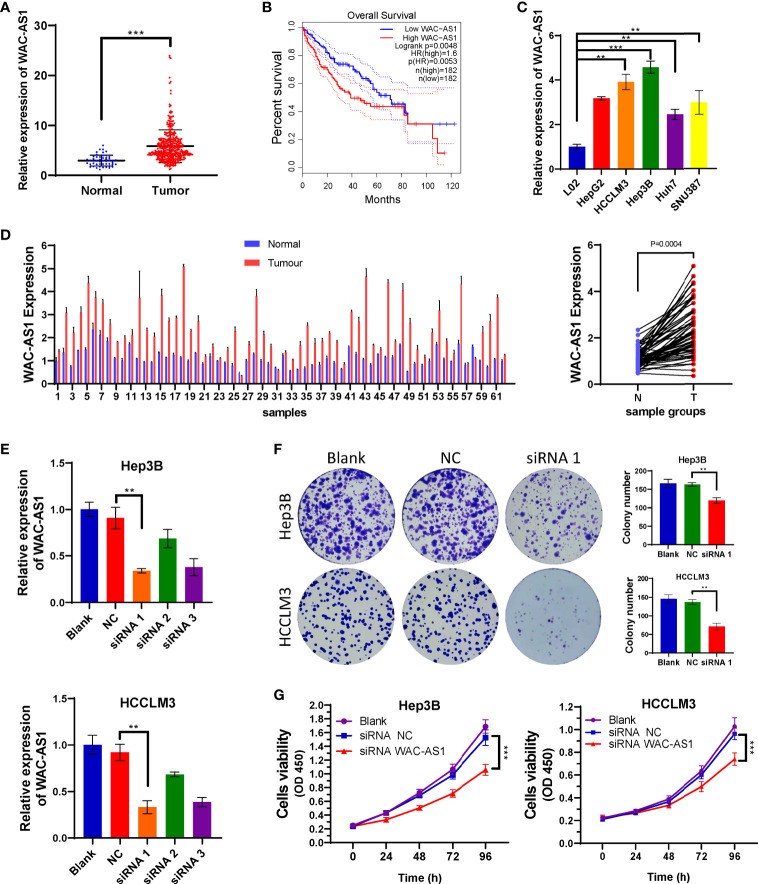
High expression of WAC-AS1 is significantly correlated with a poor prognosis and cell proliferation; **(A)** expression of WAC-AS1 in patients of the TCGA cohort; **(B)** survival curves for patients in the TCGA cohort; **(C)** expression of WAC-AS1 in cells; **(D)** expression of WAC-AS1 in patients from Zhongnan Hospital; **(E)** siRNA-mediated knockdown of WAC-AS1; **(F)** representative images of plate colony formation in Hep3B and HCCLM3 cells; **(G)** CCK-8 assay for cellular viability. ∗∗P < 0.01; ***p < 0.001.

The coefficient of WAC-AS1 in the prognostic model was high, and the expression levels of patients in the high-risk group and the low-risk group were significantly different. There is no existing research report on WAC-AS1 in tumors, so we chose WAC-AS1 for experimental verification. We analyzed the expression of WAC-AS1 in 50 normal liver tissue samples and 374 liver cancer tissue samples in the TCGA database and found that WAC-AS1 expression was significantly higher in liver cancer tissues than in normal tissues (P<0.001; **Figure 5A**). According to the median value of expression, the patients were divided into two groups. The results of survival analysis showed that patients with high WAC-AS1 expression had a significantly poorer prognosis than patients with low WAC-AS1 expression (P<0.001; **Figure 5B**). We verified this hypothesis in normal liver cell lines and liver cancer cell lines: WAC-AS1 expression was significantly higher in liver cancer cell lines (**Figure 5C**). We collected tumor samples and paracancerous samples from 62 liver cancer patients from the Biological Repositories, Zhongnan Hospital of Wuhan University and verified that WAC-AS1 was highly expressed in tumor tissues compared with adjacent normal tissues (P<0.001, **Figure 5D**). Additionally, the expression of other lncRNAs in tumor tissues and adjacent normal tissues was detected by qRT-PCR (**Figure S7**).

We next analyzed the correlation between the expression of WAC-AS1 and clinicopathologic features of patients. WAC-AS1 expression was associated with lymph node invasion (P = 0.021) and tumor diameter (P = 0.001). However, no correlation between WAC-AS1 expression and age (P = 0.602), sex (P = 0.788), HBV infection (P=0.199) or portal vein tumor thrombosis (PVTT, P = 0.073) was found (**Table 1**). We also analyzed the relative risks indicated by WAC-AS1 in the prognosis of liver cancer. The results of univariate Cox regression analysis showed that WAC-AS1 expression(P=0.004), AFP levels(P=0.002), tumor diameter (P=0.031), clinical stage (P<0.001), PVTT (P=0.009) and Lymphatic invasion (P<0.001) are correlated with prognosis. Multivariate Cox regression analysis further confirmed that the expression of WAC-AS1 is an independent prognostic factor of the survival duration of patients (P=0.007) (**Table 2**). These results indicate a significant correlation of the expression of WAC-AS1 with the prognosis of liver cancer.

**Table 1:** Correlation between WAC-AS1 expression and clinicopathologic characteristics of liver cancer patients.

Characteristics	Expression of WAC-AS1		P/χ^2^ value
	Low	High	
**Age(y)**			0.602
<65	18	20	
≥65	13	11	
**Gender**			0.788
Male	20	21	
Female	11	10	
**HBV infection**			0.199
Yes	27	23	
No	4	8	
**AFP**			0.203
<400	17	12	
≥400	14	19	
**Tumor diameter (cm)**	3.629 ± 1.6209	5.074 ± 1.8672	0.001
**TNM classification**			0.124
I	2	0	
II	11	6	
III	13	14	
IV	5	11	
**PVTT**			0.073
Yes	10	17	
No	21	14	
**Lymphatic invasion**			0.021
Yes	13	22	
No	18	9	

PVTT, Portal vein tumor thrombosis; HBV, hepatitis B virus; AFP, Alpha-fetoprotein.

**Table 2:** Univariate and multivariate Cox-regression analysis of various prognostic parameters in patients with liver cancer.

	Univariate Analysis			Multivariate Analysis		
	P	HR	95% CI	P	HR	95% CI
Age ≥ 65	0.420	1.324	0.669-2.620			
Gender - male	0.183	0.629	0.318-1.244			
HBV infection (+)	0.556	1.434	0.432-4.754			
AFP ≥ 400	0.002	3.331	1.541-7.198	0.133	1.979	0.812-4.823
Tumor diameter ≥ 4.5 cm	0.031	1.241	1.020-1.509	0.200	1.174	0.919-1.499
TNM classification(III and IV)	<0.001	3.547	1.968-6.394	<0.001	3.097	1.636-5.863
PVTT (+)	0.009	2.489	1.258-4.924	0.004	3.336	1.456-7.639
Lymphatic invasion (+)	<0.001	4.796	2.201-10.453	0.013	3.063	1.268-7.402
WAC-AS1 – high expression	0.004	1.620	1.165-2.252	0.007	1.613	1.140-2.283

HR, Hazard ratio; CI, confidence interval; +, patients with HBV infection, PVTT or lymphatic invasion.

### Knockdown of WAC-AS1 Can Affect the Proliferation and Cell Cycle of Liver Cancer Cells

**Figure 6:**
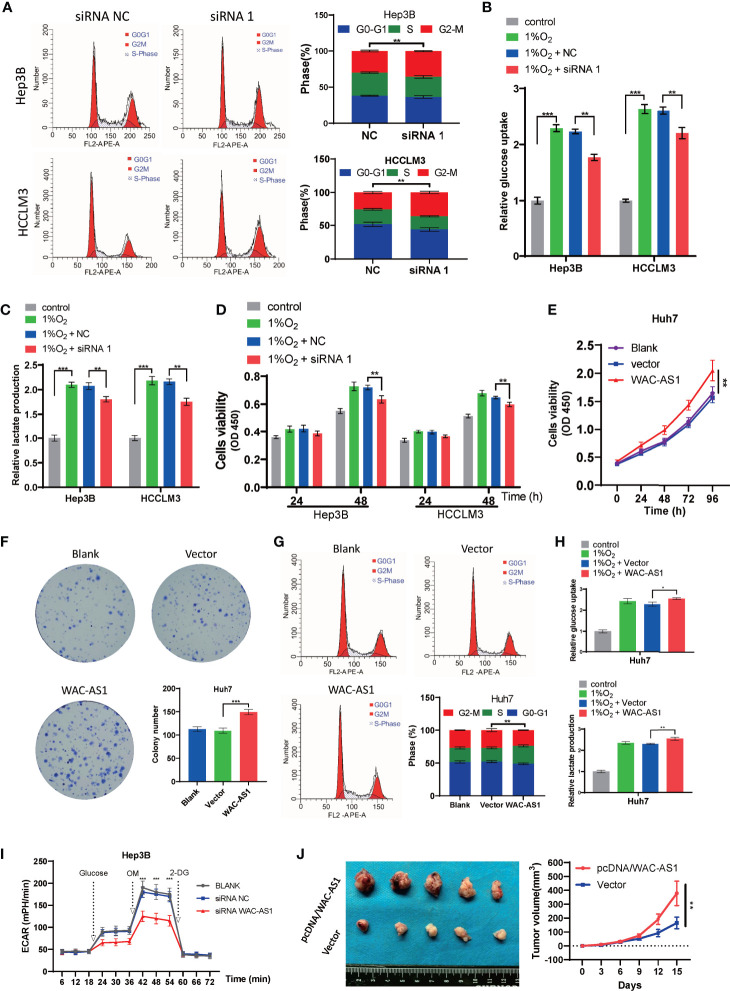
The effect of WAC-AS1 on glycolysis and the cell cycle. **(A)** cell cycle analysis was used to detect the cell cycle distribution after transfection; **(B)** glucose uptake in Hep3B and HCCLM3 cells; **(C)** lactate production detected by the lactate assay kit; **(D)** CCK-8 assay for cell viability; **(E)** cell viability of Huh7 cells after overexpression of WAC-AS1; **(F)** representative images of plate colony formation in Huh7 cells; **(G)** cell cycle analysis of Huh7 cells; **(H)** glucose uptake and lactate production in Huh7 cells; **(I)** ECAR levels in Hep3B cells; **(J)** subcutaneous xenograft tumors and growth curve. ∗P < 0.05; ∗∗P < 0.01; ***p < 0.001.

Because WAC-AS1 has the highest expression in HCCLM3 and Hep3B cell lines, we chose these two cell lines to knock down WAC-AS1 using siRNA. Among the three designed siRNA sequences, siRNA 1 had the highest knockdown efficiency (**Figure 5E**). Next, colony formation experiments and CCK-8 proliferation assay experiments were performed to evaluate the effect of WAC-AS1 on cell proliferation. The results showed that knockdown of WAC-AS1 significantly inhibited cell proliferation and cell activity (**Figures 5F, G**). When WAC-AS1 was knocked down, the cell cycle analysis results showed that the proportion of cells in the G2-M phase was significantly increased, indicating cell cycle arrest, which is consistent with the results of cell proliferation experiments and GSEA (**Figure 6A**).

### Knockdown of WAC-AS1 Affects Glycolysis in Tumor Cells

HCCLM3 and Hep3B cells were exposed to 1% O_2_ to simulate hypoxic conditions for cell culture. Then, under hypoxic conditions, the lactate concentration and glucose uptake of HCCLM3 and Hep3B cells that were treated with siWAC-AS1 and siRNA-NC were detected. Under hypoxic conditions, glucose uptake and lactic acid production of cells were significantly increased when WAC-AS1 was knocked down (**Figures 6B, C**). This shows that the knockdown of WAC-AS1 can inhibit glycolysis in cancer cells under hypoxic conditions. In addition, the CCK-8 assay performed under the same conditions showed that the viability of tumor cells was significantly increased under hypoxic conditions, but the knockdown of WAC-AS1 decreased the viability of tumor cells (**Figure 6D**). These data indicate that WAC-AS1 may inhibit HCC cell proliferation by inhibiting glycolysis under hypoxic conditions.

### Overexpression of WAC-AS1 Promotes Tumor Cell Proliferation and Glycolysis

Because the expression of WAC-AS1 in Huh7 cells is relatively low, we overexpressed WAC-AS1 in the Huh7 cell line. The WAC-AS1 plasmid and blank plasmid were transfected into Huh7 cells, and the CCK-8 assay was used to detect cell viability. Compared with the control group, the cell viability of WAC-AS1-overexpressing cells was significantly improved (**Figure 6E**). After WAC-AS1 overexpression, the proliferation ability of cells was significantly enhanced (**Figure 6F**), and the proportion of cells in the S phase increased, suggesting that cell proliferation was accelerated (**Figure 6G**). Under the same hypoxic conditions, the glycolysis efficiency of the cells after WAC-AS1 overexpression was tested. We found that the overexpression of WAC-AS1 promoted glucose uptake and lactate production in tumor cells compared with that in tumor cells of the control group (**Figure 6H**). Furthermore, we measured the effect of WAC-AS1 on ECAR. Knockdown of WAC-AS1 significantly reduced ECAR levels in Hep3B cells compared to control cells (**Figure 6I**). Conversely, overexpression of WAC-AS1 significantly increased ECAR levels in Huh7 cells (**Figure S8**). We performed subcutaneous tumor experiment by Huh7 cells in mice. The result showed that pretreatment with pcDNA/WAC-AS1 significantly promoted tumor-forming capacity of Huh7 *in vivo* (**Figure 6J**). The animal study was reviewed and approved by The Animal Ethical Committee of Zhongnan Hospital of Wuhan University.

### Verification of the Co-Expression Relationship Between WAC-AS1 and GR Genes

**Figure 7:**
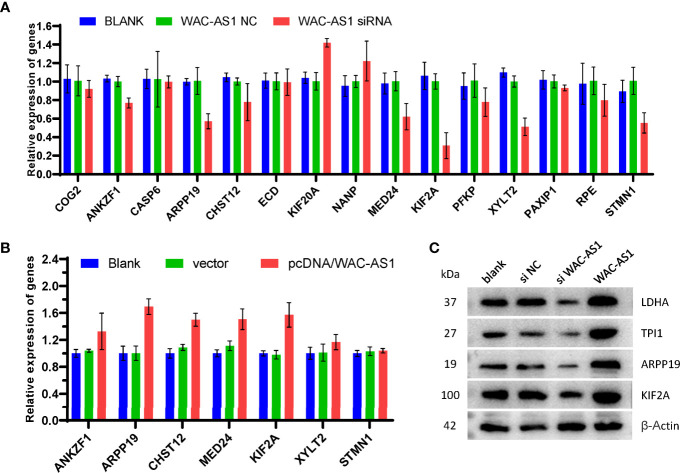
Verification of co-expression relationships. **(A)** expression of GR genes in HCCLM3 cells treated with siRNA; **(B)** expression of GR genes in HCCLM3 cells after overexpression of WAC-AS1; **(C)** expression of GR genes detected by western blotting.

The results of bioinformatics analysis showed that WAC-AS1 has a co-expression relationship with a variety of GR genes (ANKZF1, ARPP19, CASP6, CHST12 and PAXIP1). When WAC-AS1 was knocked down in HCCLM3 cells, the expression of ANKZF1, ARPP19, CHST12, MED24, KIF2A, XYLT2 and STMN1 also decreased significantly (**Figure 7A**). When WAC-AS1 was overexpressed, the expression levels of ARPP19, CHST12, MED24 and KIF2A (among the 7 genes) were upregulated (**Figure 7B**). Among the 4 upregulated GR genes, ARPP19 is related to the cell cycle (23). The GSEA results for both ARPP19 and WAC-AS1 showed the enrichment of cell cycle pathways. Therefore, we believe that WAC-AS1 may regulate the cell cycle through ARPP19 and may also regulate glycolysis. When WAC-AS1 was knocked down or overexpressed, the protein levels of ARPP19 and KIF2A changed correspondingly. The markers of glycolysis LDHA and TPI1 also showed the same trend of change (**Figure 7C**).

### WAC-AS1 Regulated ARPP19 by Sponging miR-320d

**Figure 8:**
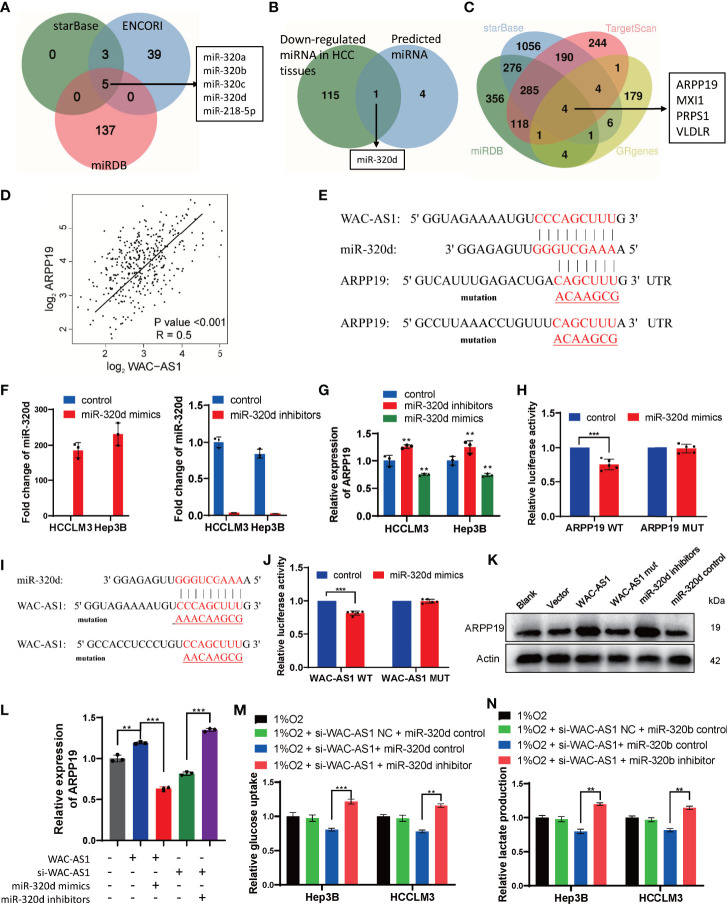
WAC-AS1 regulated ARPP19 by sponging miR-320d. **(A)** venn diagram showing the predicted target genes of WAC-AS1 from databases (miRDB, ENCORI, and starBase); **(B)** screening out target genes with differences analysis; **(C)** venn diagram showing the predicted target genes of miR-320d; **(D)** the correlation analysis between WAC-AS1 and ARPP19 using data from TCGA database; **(E)** the wild-type and the mutated sequences of the ARPP19 mRNA 3’-UTR (mutation site: red); **(F)** the levels of miR-320d after miR-320d mimics and miR-320d inhibitor were transfected; **(G)** expression of the ARPP19 after treated with miR-320d mimics or inhibitor; **(H)** the luciferase activity in luciferase reporter plasmid containing wild-type ARPP19 and mutated ARPP19 co-transfected with miR-320d mimics or negative control; **(I)** the wild-type and the mutated sequences of the WAC-AS1; **(J)** the luciferase activity in luciferase reporter plasmid containing wild-type WAC-AS1 and mutated WAC-AS1 co-transfected with miR-320d mimics or negative control; **(K)** the protein levels of ARPP19 were regulated by WAC-AS1 and miR-320d; **(L)** ARPP19 expression in differentially treated cells; **(M)** glucose uptake in Hep3B and HCCLM3 cells; **(N)** lactate production detected by the lactate assay kit. ∗∗P < 0.01; ***p < 0.001.

The correlation analysis between WAC-AS1 and GR genes was based on RNA sequencing data, so we assumed that regulation might occur post-transcriptionally, for example, through microRNA. To verify this hypothesis, starBase, ENCORI and miRDB were used to predict the target miRNA of WAC-AS1(**Figure 8A**). Through the difference analysis to further narrow down the scope, it was screened out that miR-320d may be the target of WAC-AS1 (**Figure 8B**). The target genes of miRNA-320d were screened by starBase, miRDB and TargetScan. In the results, 4 genes were glycolysis-related genes, including ARPP19 (**Figure 8C**). In the TCGA database, the expression levels of WAC-AS1 and ARPP19 were positively correlated (**Figure 8D**), so we hypothesize that WAC-AS1 may regulate ARPP19 by sponging miR-320d. We predicted the binding sites between RNAs, and synthesized wild-type and mutant ARPP19 plasmids (ARPP19 WT and MUT) (**Figure 8E**). We synthesized miR-320d mimics and miR-320d inhibitor, which had been verified to have high overexpression and knockdown efficiency (**Figure 8F**). The expression of ARPP19 was regulated by miR-320d (**Figure 8G**). The dual luciferase experiment showed that luciferase activity was suppressed by miR-320d mimics, indicating that miR-320d can directly bind to ARPP19 and promote its degradation (**Figure 8H**). A plasmid with the mutant sequence of WAC-AS1(WAC-AS1 MUT) was synthesized (**Figure 8I**), and the combination of miRNAd and WAC-AS1 was confirmed through dual luciferase experiments (**Figure 8J**). Knockdown of miR-320d or overexpression of WAC-AS1 increased the expression of ARPP19, while WAC-AS1 MUT did not regulate the expression of ARPP19 (**Figure 8K**). Overexpression of WAC-AS1 can upregulate the mRNA level of ARPP19, and this regulation can be reversed by miR-320d mimics. The downregulation of ARPP19 in cells treated with siRNA of WAC-AS1 can be reversed by miR-320d inhibitor, indicating that WAC-AS1 regulates ARPP19 through miR-320d (**Figure 8L**). Under hypoxic conditions, WAC-AS1 can regulate the glucose uptake and lactate production of cells, and this regulation can also be reversed by miR-320d (**Figures 8M, N**).

## Discussion

HCC is one of the most common cancers in the world. Although recent studies have made great progress in the treatment, prognosis and diagnosis of HCC, its morbidity and mortality rates are still high (24). The treatment of HCC faces many challenges, one of which is that the prognosis of patients is difficult to predict, which brings difficulties in clinical management and clinical decision-making (25, 26). Increasing evidence shows that many lncRNAs have important biological functions and can participate in various physiological and pathological processes, including tumorigenesis, development, metabolism and tumor immunity (17, 27, 28). Among them, lncRNAs are closely related to tumor glycolysis (29, 30). However, only a few lncRNAs have been reported to regulate glycolysis in tumors. In view of the extensive and important physiological functions of lncRNAs, there should be many lncRNAs that can regulate glycolysis that have not yet been discovered. Therefore, we screened lncRNAs that may have the potential to regulate tumor glycolysis through bioinformatics methods, which can guide future research, and based on this, we constructed a prognostic model for liver cancer patients.

We screened 502 lncRNAs related to glycolysis using co-expression analysis. Because the data we analyzed were at the RNA expression level, some GR lncRNAs may not have been screened out. For example, lncRNA LINRIS can affect tumor glycolysis by stabilizing the IGF2BP2 protein, but there is no co-expression relationship between LINRIS and IGF2BP2 at the RNA level (30). According to survival analysis, 36 DE lncRNAs that are related to glycolysis and prognosis were further screened. Among them, SNHG3 has been reported to be related to tumor glycolysis, but the role of most other lncRNAs in glycolysis has not been reported (31). Therefore, our results can provide directions for future research, and the mechanism of action of these 36 lncRNAs is worthy of in-depth research in the future.

The prognostic model based on 8 lncRNAs established by LASSO regression analysis was verified to have good predictive performance. The prognostic model can predict patient prognostic risk. The established nomogram can be conveniently applied in the clinic to predict the 1-, 2-, 3- and 5-year survival rates of patients. Survival prediction and risk rating can help with clinical management and treatment decision-making. For example, transcatheter arterial chemoembolization (TACE) treatment after liver tumor resection can facilitate local tumor control and prolong progression-free survival, but it has certain side effects. Therefore, whether TACE should be used in patients with highly differentiated liver cancer or low-stage patients is controversial (32, 33). According to the model we established, the prognostic risk of the patient can be judged, and then we can choose whether to use TACE and other adjuvant therapies.

The GSEA results for the lncRNAs in the risk score showed that they were frequently enriched in cell cycle-related pathways. In subsequent experiments, we also discovered the effect of WAC-AS1 on the cell cycle. The WAC-AS1-related glycolysis gene ARPP19 also plays an important role in regulating the cell cycle (34, 35). According to previous studies, the enhancement of tumor glycolysis metabolism can promote cell proliferation, and the inhibition of glycolysis can induce cell cycle arrest (36, 37). Therefore, the enrichment of cell cycle pathways in the GSEA results helped to verify the glycolysis-regulating effects of lncRNAs. The lncRNAs in the risk model may affect the cell cycle by regulating glycolysis, thereby promoting cell proliferation and disease progression.

In the past ten years, immunotherapy has gradually become a major systemic treatment for cancer. In recent years, the role of metabolic reprogramming of cells in the tumor microenvironment on tumor immunity has gradually become a research hotspot (38). Due to the disorder of tumor cell metabolism, the immune cells infiltrating the tumor induce a metabolic emergency, which leads to the impairment of the antitumor immune response (39). Therefore, we tried to explore the relationship between glycolysis, immune cell infiltration and immune function. We found that the risk score and GR lncRNAs are related to immune-related functions and the abundance of a variety of immune cells. Among them, MSC-AS1 is associated with various immune functions, such as CD8 T cells, B cells, macrophages, antigen presentation and the IFN immune response. MSC-AS1 is reported to be related to tumor proliferation, apoptosis and immunity (40–42), but its relationship and mechanism with tumor immunity have not been studied in depth. Therefore, researching the lncRNAs we have screened in the future may help to reveal the regulatory mechanism of tumor immunity and reveal new immunotherapy targets.

In our research, we identified 8 lncRNAs that are highly related to GR genes and verified the biological functions of WAC-AS1 through experiments. Several studies have reported the carcinogenic effects of lncRNAs in the model. For example, MIR210HG is associated with a poor prognosis in HCC patients and exerts a carcinogenic effect (43). SNHG12 can promote the development and metastasis of HCC (44). However, few studies have shown that there is a link between GR lncRNAs, glycolysis and immune regulation in cancer. Our analysis shows that multiple lncRNAs, such as MIR210HG, WAC-AS1, and MSC-AS1, are involved in the relationship between glycolysis, tumor progression, immune cell infiltration and the immune response.

In this study, we identified miR-320d as a key molecule in the process by which WAC-AS1 regulates ARPP19. Furthermore, we demonstrated that WAC-AS1 functioned as a competing endogenous RNA (ceRNA) to affect miR-320d activity and regulate the miR-320d target gene ARPP19. It has been reported that miR-320a, another member of the miR-320 family, can increase glycolysis (45). We discovered for the first time that miR-320d can regulate glycolysis. The results of the dual luciferase assay showed that miR-320d binds to the 3’UTR of ARPP19 directly and negatively regulates the expression of ARPP19. miR-320d shares high nucleic acid sequence similarity with other members of the miR-320 family. Therefore, miR-320b and miR320c may also have the potential to regulate tumor glycolysis, and further research is needed.

This study has some shortcomings. One limitation is the lack of a large number of patients with RNA sequencing data and follow-up data to verify the classification performance and survival prediction performance of our prediction model. The second limitation is the lack of proteomics detection data; therefore, the changes in the protein levels of GR genes have not been included in the bioinformatics analysis. Third, the lncRNAs we screened have not been previously researched, so their regulatory mechanisms need further study.

## Data Availability Statement

The datasets presented in this study can be found in online repositories. The names of the repository/repositories and accession number(s) can be found in the article/**Supplementary Material**.

## Ethics Statement

The studies involving human participants were reviewed and approved by the Ethics Committee of Zhongnan Hospital. The patients/participants provided their written informed consent to participate in this study. The animal study was reviewed and approved by the Animal Ethical Committee of Zhongnan Hospital of Wuhan University.

## Author Contributions

XX and HZ designed the study, conducted the experiments and literature search, and generated the figures. PX performed the dual-luciferase assay. YZ, JL, and KX wrote and edited the manuscript. YY helped perform the analysis with constructive discussions. All authors contributed to the article and approved the submitted version.

## Funding

Our work was supported by the Research Fund of the Health Commission of Hubei Province (WJ2021M255); the Cancer Research and Translational Platform Project of Zhongnan Hospital of Wuhan University (ZLYNXM202004); the Translational Medicine and Interdisciplinary Research Joint Fund Project of Zhongnan Hospital of Wuhan University (ZNJC201918); a grant from the National Key Research and Development Program of China (SQ2019YFC200078/02).

## Conflict of Interest

The authors declare that the research was conducted in the absence of any commercial or financial relationships that could be construed as a potential conflict of interest.

## Publisher’s Note

All claims expressed in this article are solely those of the authors and do not necessarily represent those of their affiliated organizations, or those of the publisher, the editors and the reviewers. Any product that may be evaluated in this article, or claim that may be made by its manufacturer, is not guaranteed or endorsed by the publisher.

## Abbreviations

lncRNA, Long non-coding RNA; TCGA, The Cancer Genome Atlas; WAC-AS1, WAC antisense RNA 1; ARPP19, cAMP regulated phosphoprotein 19; GR genes, Glycolysis-related genes; GR lncRNAs, Glycolysis-related lncRNAs; DEgenes, Differentially expressed genes; ROC, Receiver operating characteristic; AUC, Area under the curve; PCA, Principal component analysis; CCK-8, Cell Counting Kit-8.
