# Supplementary figures and images for: Identification of Glycolysis-Related lncRNAs and the Novel lncRNA WAC-AS1 Promotes Glycolysis and Tumor Progression in Hepatocellular Carcinoma
Source: Front Oncol. 2021 Aug 30;11:733595. doi: 10.3389/fonc.2021.733595 (PMC8437343; doi:10.3389/fonc.2021.733595)

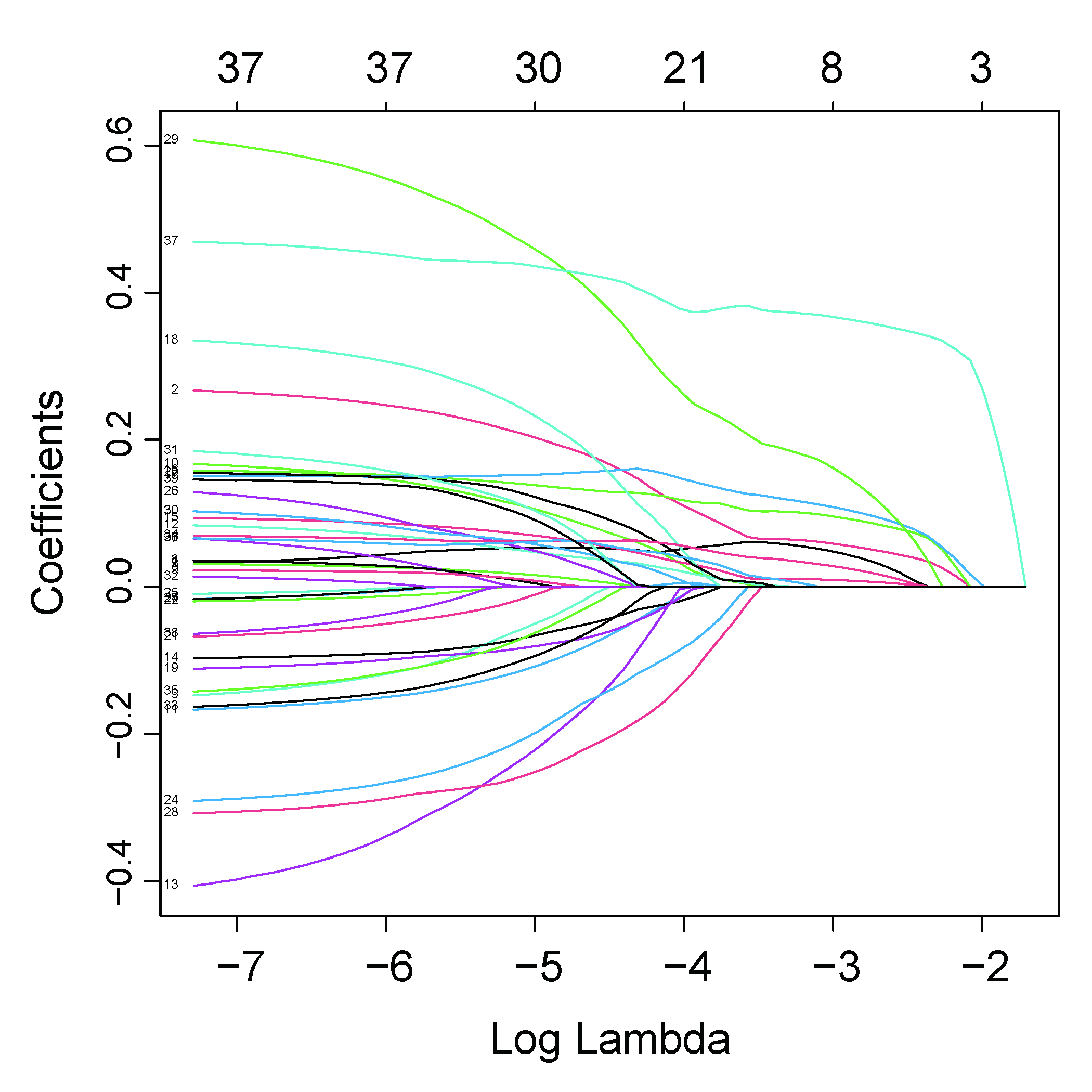

Supplement: Supplementary file 1 [file DataSheet_1.zip › Supplementary material/Figure S1.tif]

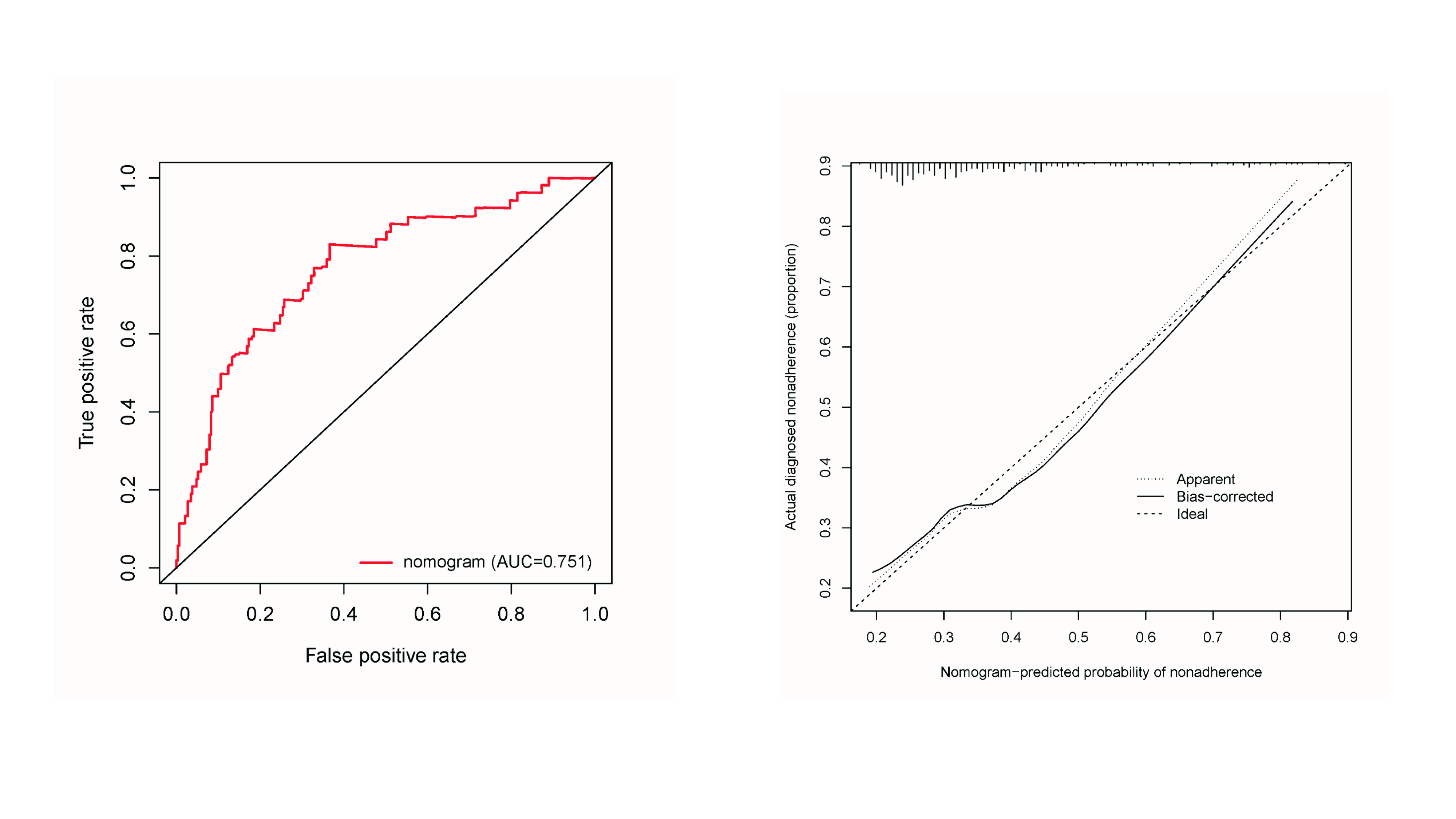

Supplement: Supplementary file 1 [file DataSheet_1.zip › Supplementary material/Figure S2.tif]

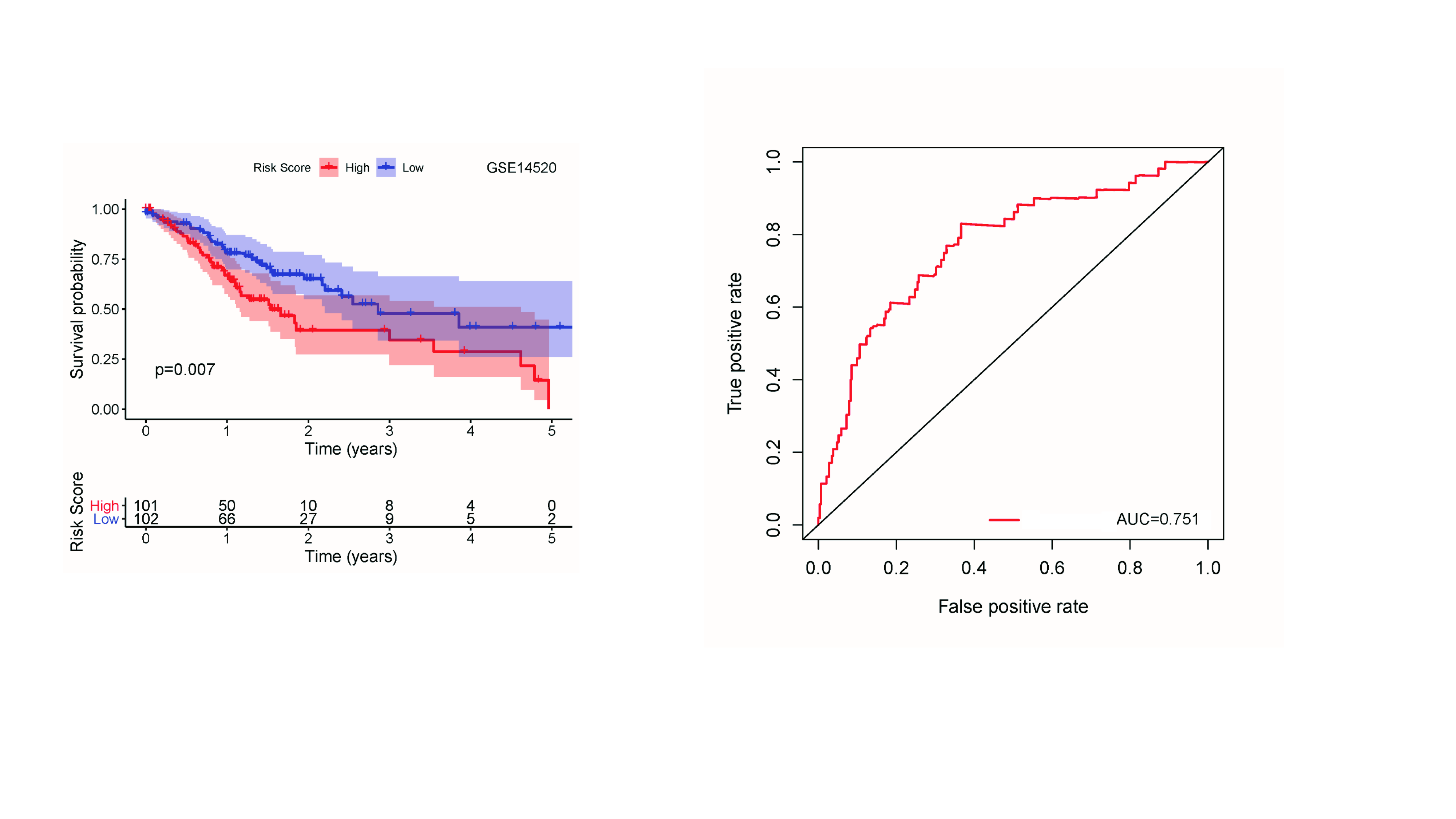

Supplement: Supplementary file 1 [file DataSheet_1.zip › Supplementary material/Figure S3.tif]

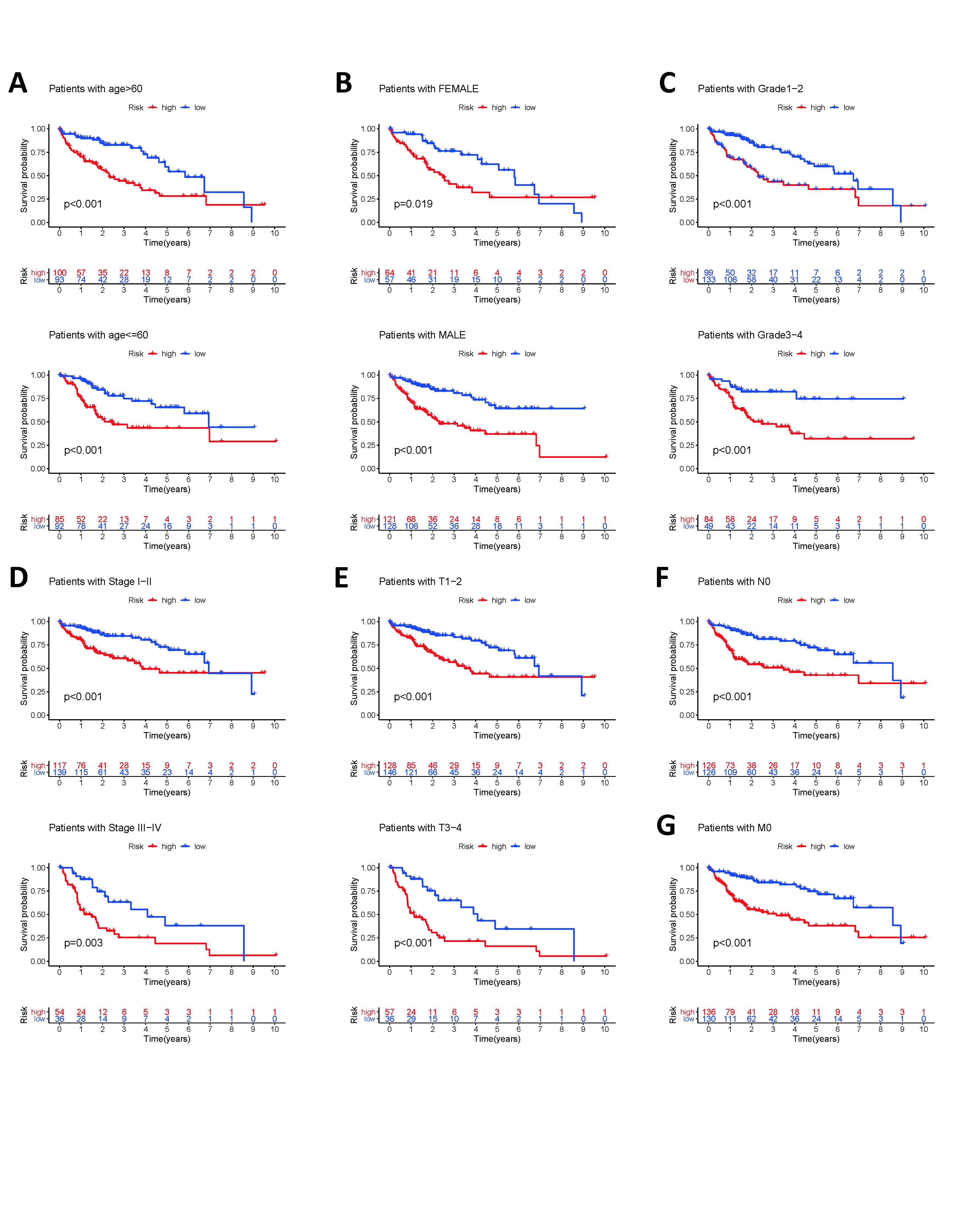

Supplement: Supplementary file 1 [file DataSheet_1.zip › Supplementary material/Figure S4.tif]

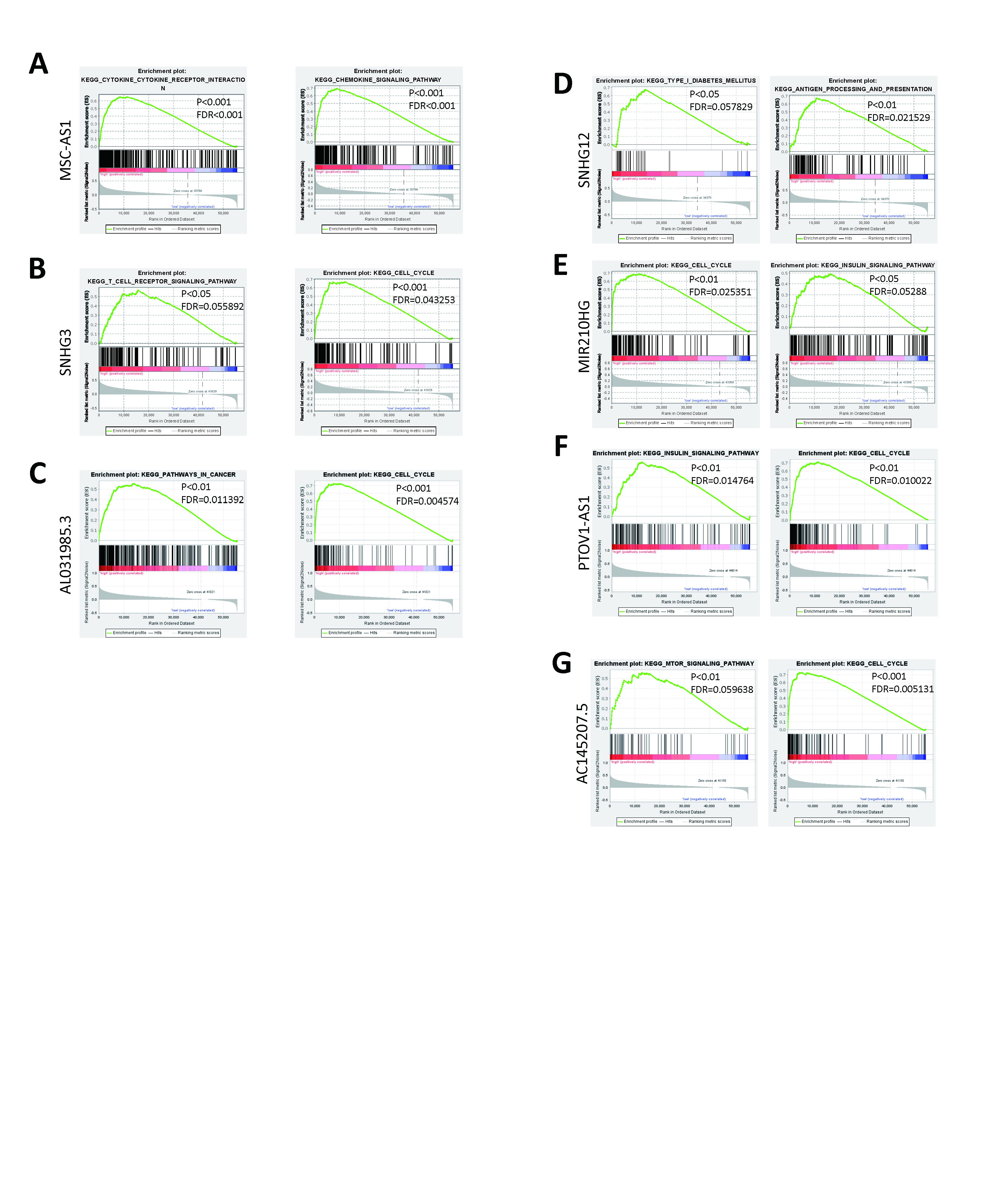

Supplement: Supplementary file 1 [file DataSheet_1.zip › Supplementary material/Figure S5.tif]

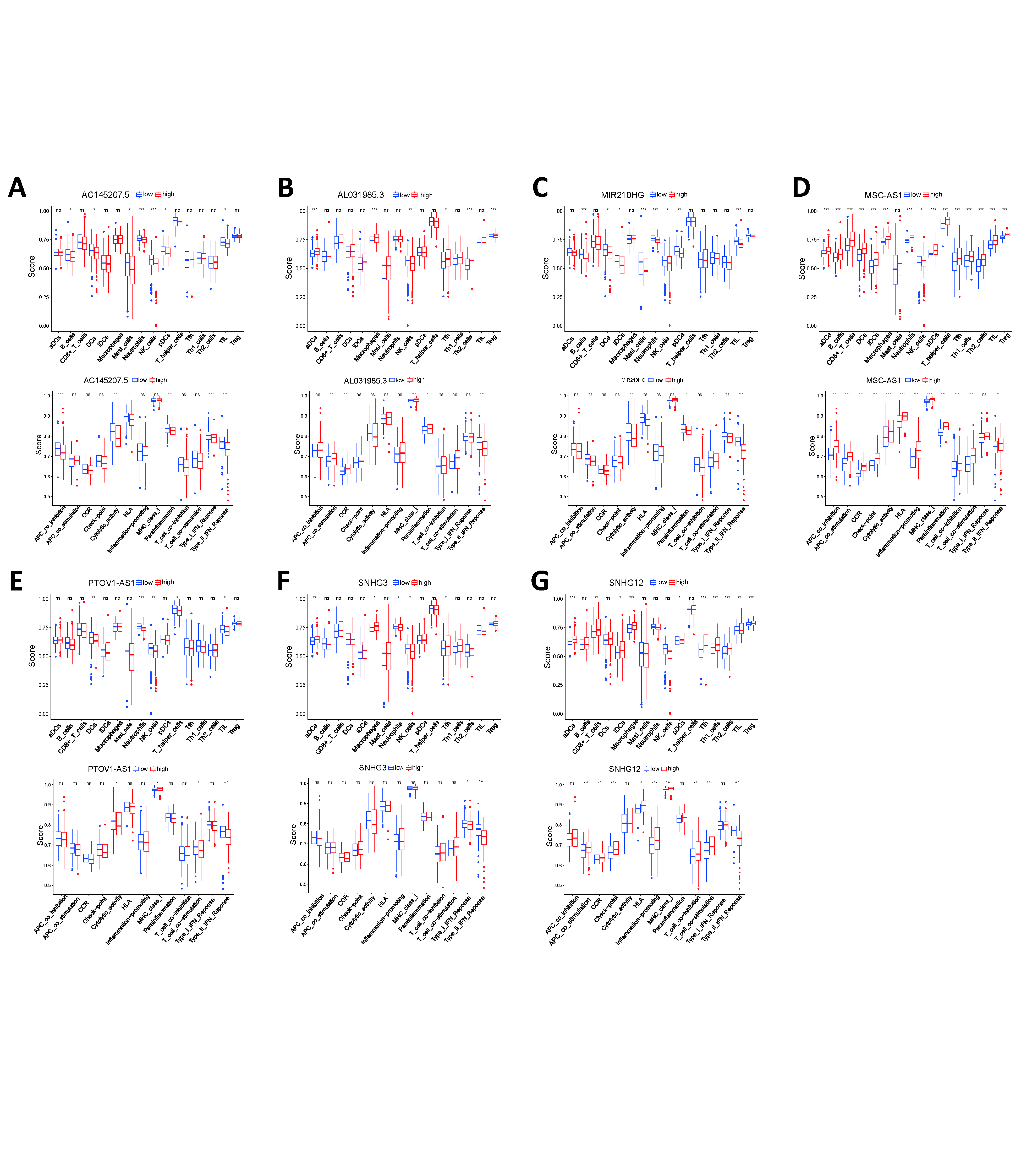

Supplement: Supplementary file 1 [file DataSheet_1.zip › Supplementary material/Figure S6.tif]

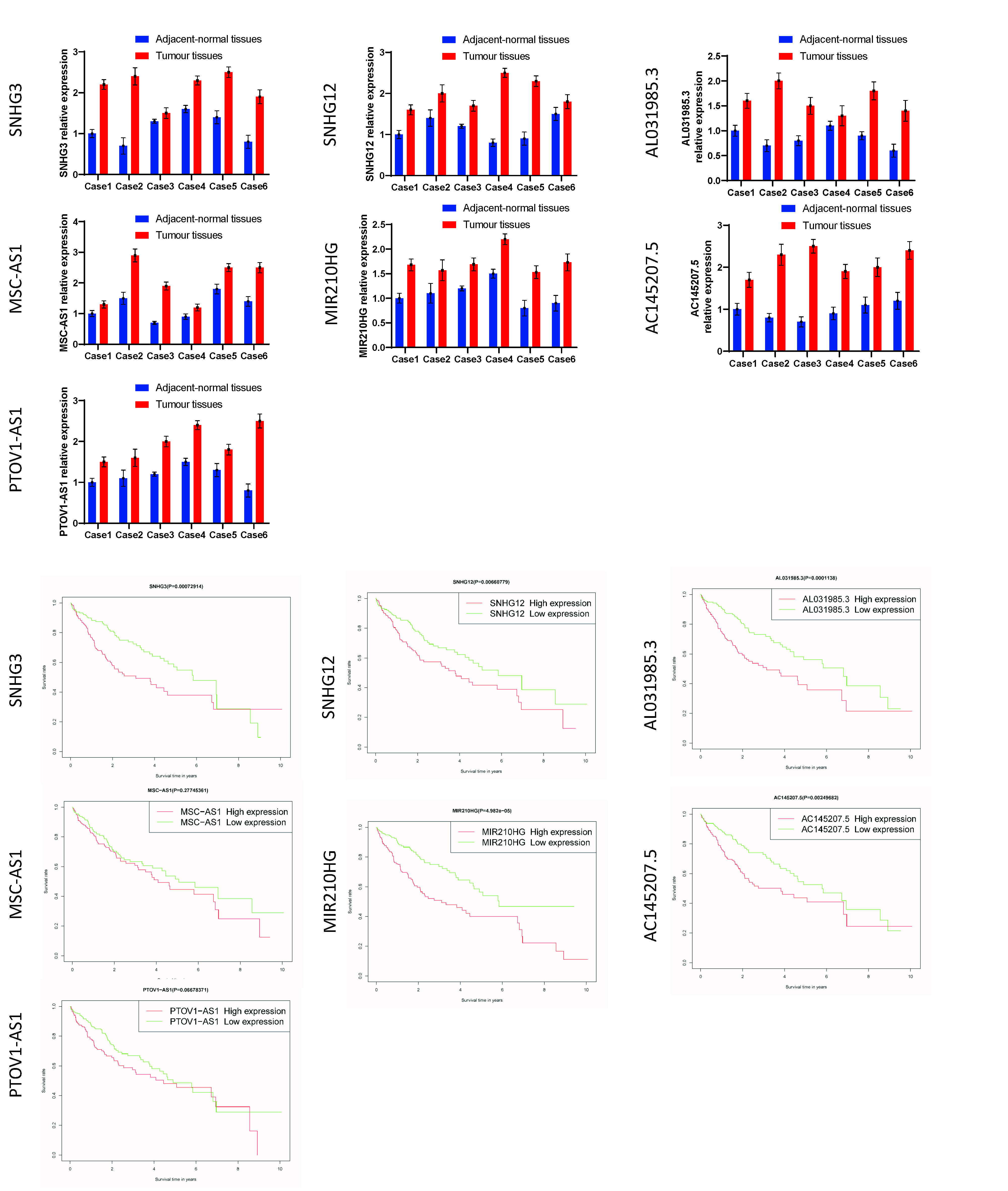

Supplement: Supplementary file 1 [file DataSheet_1.zip › Supplementary material/Figure S7.tif]

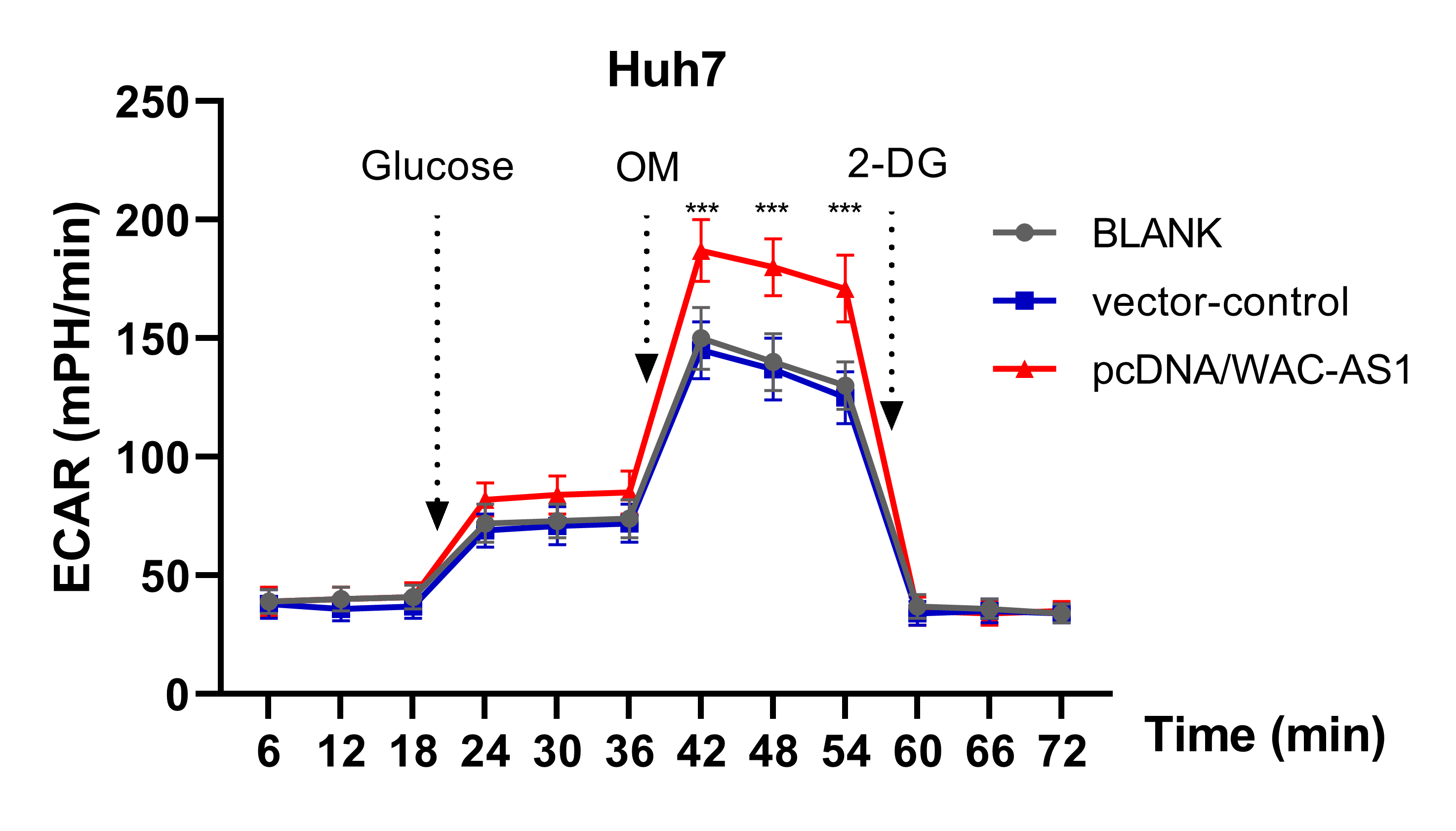

Supplement: Supplementary file 1 [file DataSheet_1.zip › Supplementary material/Figure S8.tif]
